# Transformer based neural network for daily ground settlement prediction of foundation pit considering spatial correlation

**DOI:** 10.1371/journal.pone.0294501

**Published:** 2023-11-20

**Authors:** Xiaofeng Wu, Song Yang, Di Zhang, Liang Zhang

**Affiliations:** 1 College of Civil Engineering and Architecture, Zhejiang University, Hangzhou, Zhejiang, China; 2 Advanced Potential Talent Center, Hangzhou Urban Construction & Investment Group Co., Ltd., Hangzhou, Zhejiang, China; 3 Department of Chief Engineer, Hangzhou CBD Construction and Development Co., Ltd., Hangzhou, Zhejiang, China; 4 Housing Construction Project Department, Hangzhou CBD Construction and Development Co., Ltd., Hangzhou, Zhejiang, China; University 20 Aout 1955 skikda, Algeria, ALGERIA

## Abstract

Deep foundation pit settlement prediction based on machine learning is widely used for ensuring the safety of construction, but previous studies are limited to not fully considering the spatial correlation between monitoring points. This paper proposes a transformer-based deep learning method that considers both the spatial and temporal correlations among excavation monitoring points. The proposed method creates a dataset that collects all excavation monitoring points into a vector to consider all spatial correlations among monitoring points. The deep learning method is based on the transformer, which can handle the temporal correlations and spatial correlations. To verify the model’s accuracy, it was compared with an LSTM network and an RNN-LSTM hybrid model that only considers temporal correlations without considering spatial correlations, and quantitatively compared with previous research results. Experimental results show that the proposed method can predict excavation deformations more accurately. The main conclusions are that the spatial correlation and the transformer-based method are significant factors in excavation deformation prediction, leading to more accurate prediction results.

## Introduction

Deep foundation pit is an essential part of urban construction, which involves various fields such as real estate, municipal, and transportation. As a fundamental infrastructure of modern urban construction, excavation pit engineering has significant importance for urban development. However, soil settlement and ground deformation problems often accompany the construction process of excavation pit engineering, which may cause adverse effects on surrounding buildings, roads, and underground pipelines. Therefore, it is crucial to control and predict the deformation of excavation pits for safety [1–3].

Various methods have been used to predict the settlement of Deep foundation pits, such as finite element-based methods, empirical methods, and machine learning-based methods. Traditional methods, such as empirical methods and finite element-based methods, have achieved certain results in different scenarios. However, accuracy and boundary sensitivity issues still exist in practical applications [4–7]. Machine learning methods have received considerable attention in recent years, which can consider multiple factors and improve prediction accuracy. In the prediction of excavation pit deformation, many scholars have applied machine learning methods [8–11], such as neural networks [12, 13], support vector machines [14, 15], and random forests [16]. Nejad and Jaksa [17] used a supervised learning algorithm to simulate load settlement based on CPT data and found that too many input variables could seriously affect its application ability. Cao et al. [18] discussed the influence of different input variables on settlement based on parameter sensitivity analysis. Feng et al. [19] examined the impact of factors such as excavation depth and the number of internal supports on foundation pit settlement using the BP algorithm. Zhang et al. [20] considered the influence of various factors (O’Rourke, [21]; Seo et al., [22]) and used the ANN algorithm to predict excavation pit settlement.

Temporal correlation is considered to be one of the crucial factors among various factors because settlement prediction is a complex and dynamic engineering issue. The dynamic process of construction can result in nonlinear, intermittent fluctuations in the deformation of the excavation pit over time. Consequently, scholars have recognized the deep foundation pit settlement is a time series problem for a long time [23–30]. Xie and Pan [31] developed a backpropagation neural network (BPNN) to predict the ground settlement of the excavation pit in the next stage based on the monitoring settlement data in the previous four stages. Qiao et al. [32] focused on developing an ANN-based method that considers the influence of time series by using real-time settlement monitoring data as ANN input to predict daily ground settlement. Furthermore, there has been an increasing focus on the correlation among monitoring points in recent studies. For instance, Luo et al. [33] conducted deformation predictions by incorporating various input features such as horizontal displacement, longitudinal displacement, and combined displacements in the time domain. They also utilized adjacent monitoring data from the following day to enhance their predictions. The results demonstrated the significance of spatial correlation derived from neighboring monitoring data in improving the accuracy of deformation predictions. However, spatial correlation is not given enough consideration when considering the settlement prediction of the foundation pit as a series prediction problem.

Various types of neural network architectures have been developed to model and predict time series data. Among these, recurrent neural networks (RNNs) and long short-term memory (LSTM) networks have been widely used and shown to achieve good performance in various applications [34, 35]. RNNs and LSTMs are designed to handle sequential data by maintaining a hidden state that summarizes the previous input sequence, which is then used as input for the next step. However, in long sequences, gradient vanishing and exploding problems can occur, which makes it difficult to capture long-term dependencies. Moreover, RNNs and LSTMs are sequential models and do not capture global context information, which limits their ability to capture complex patterns in time series data. In contrast, the transformer network is a non-sequential model that can capture global context information by attending to all the input time steps simultaneously [36]. The key idea of the transformer network is the self-attention mechanism, which allows the model to weigh the importance of different time steps based on their relevance to the prediction task. The Transformer network has been applied to various time series tasks, including language modeling, machine translation, and speech recognition, and has achieved state-of-the-art performance in many benchmarks, thanks to its ability to capture long-range dependencies and handle sequential data effectively. Inspired by this, compared to the traditional LSTM and RNN, the transformer’s attention mechanism could be leveraged to capture the temporal and spatial correlations among monitoring points in the context of settlement prediction. It is been noticed that traditional time series methods (RNN or LSTM) often struggle to capture complex and non-linear relationships in geotechnical data. The attention mechanism of the transformer allows the model to dynamically weigh the importance of different monitoring points and their temporal sequences, facilitating a more comprehensive understanding of the data patterns [37, 38].

In summary, existing methods can be categorized into two types: (1) approaches that do not take into account temporal correlation, as demonstrated by Nejad and Jaksa [17], Cao et al. [18]; (2) approaches that consider temporal correlation but often overlook spatial correlation, as illustrated by Xie and Pan [31], Qiao et al. [32], and Luo et al. [33]. In this study, the significance of fully considering spatial correlation is investigated using a time series model, an aspect that has been mentioned but not thoroughly investigated by Luo et al. [33]. While the prevailing time series methods commonly rely on LSTM or RNN as the underlying network architecture [39, 40], this paper proposes a transformer-based deep learning method (DFPTransformer) that considers both the spatial and temporal correlations among excavation monitoring points. All excavation monitoring points are collected into a vector instead of being treated as separate data points, creating a dataset that is convenient for the model to consider the correlation between any monitoring points. The proposed deep learning method is based on the attention mechanism, which can handle the spatial correlations among excavation monitoring points and incorporate temporal sequence information for prediction. To verify the accuracy of the model, we compare it with a machine learning method that only considers temporal correlations without considering spatial correlations, and quantitatively compare it with previous research results. Experimental results show that the proposed method can predict excavation deformations more accurately. The rest of this paper is organized as follows. Methodology section introduces the proposed network. In case study section, a deep foundation pit project is described along with the application of the proposed method, and the results and discussions of the project are presented. Finally, we conclude the paper in the last section.

## Methodology

This paper presents a deep learning algorithm along with a standard dataset comprising monitoring data from the excavation process of a deep foundation pit. Since it is a series problem, the input data has a dimension of [*sequence_in*, *input_dim*], and the output has a dimension of [*input_dim*]. The *sequence_in* refers to the number of sequences, whereas *input_dim* refers to the number of monitoring points. This implies that all observation values of the monitoring points at a given time are collected into a single vector.

### Proposed method

**Fig 1:**
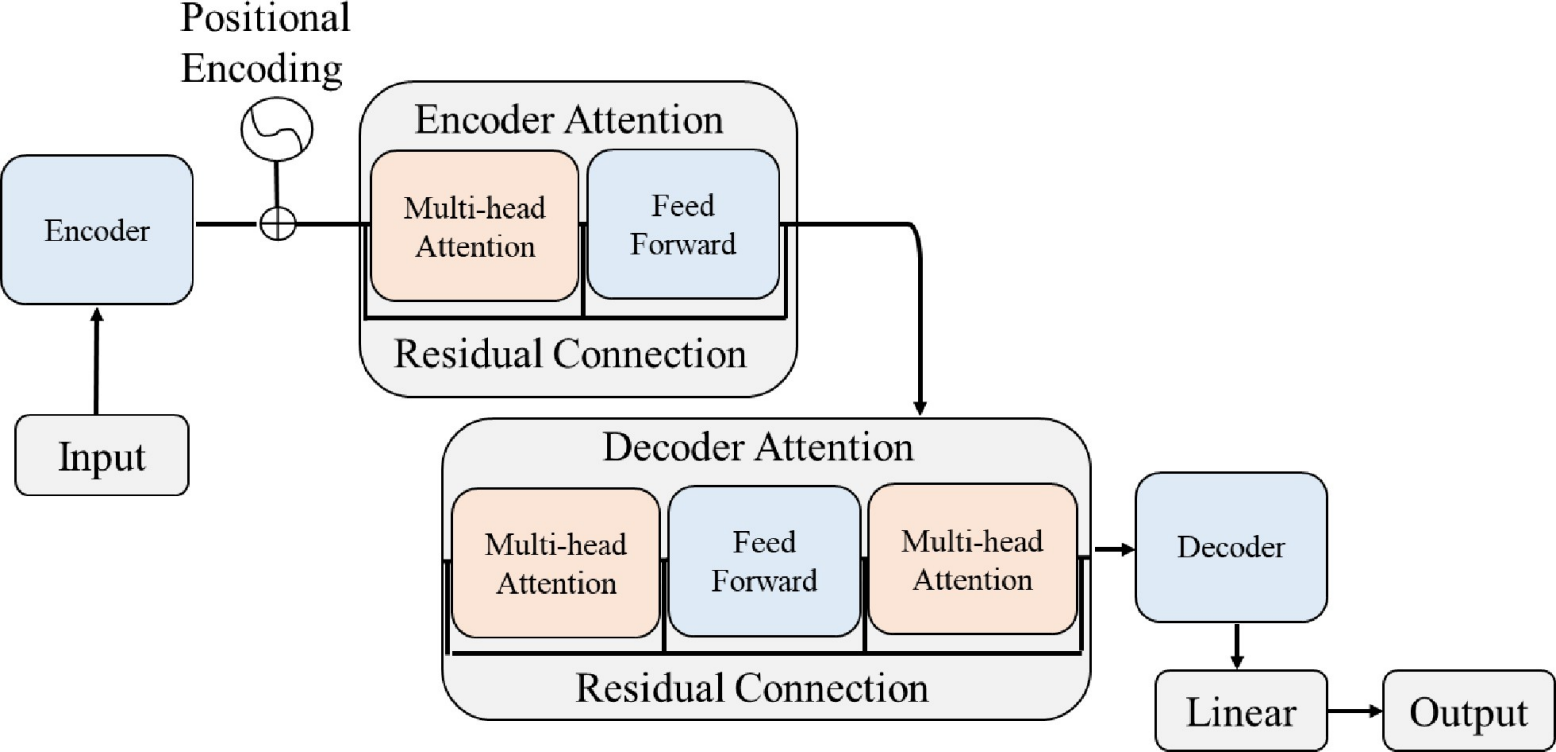
Proposed network: DFPTransformer.

Fig 1 shows a transformer-based neural network that is used for forecasting time-series deep foundation pit settlement, named DFPTransformer. It consists of an encoder, a decoder, and multiple attention mechanisms in Encoder-Attention and Decoder-Attention that allow the network to learn temporal dependencies and patterns in the input data. The architecture of the DFPTransformer network is based on the Transformer model proposed by Vaswani et al. [36]. The Transformer model is a self-attention based neural network that was originally designed for natural language processing tasks. However, it has also been successfully applied to other domains, such as time-series forecasting.

In the DFPTransformer network, the input data is first passed through an encoder, which consists of a linear layer, a ReLU activation function, and a dropout layer. The encoder is responsible for transforming the input data into a high-dimensional representation that can be easily processed by the attention mechanisms. After the input data is encoded, the positional encoding is added to the input. Positional encoding is a learnable parameter that allows the network to encode the temporal information of the input data. The positional encoding is added to the input by concatenating it with the output of the encoder.

The next step is to apply the encoder self-attention mechanism to the encoded input. The encoder self-attention mechanism is used to capture the temporal dependencies within the input sequence. The self-attention mechanism takes the encoded input as the input queries, keys, and values, and outputs the attention weights and the output feature maps. The output feature maps are then fed through a feed-forward network consisting of a linear layer, a ReLU activation function, and a dropout layer. This feed-forward network is responsible for adding non-linearity to the output feature maps. Given the limited amount of data will prevent the deep learning model from learning the relationship between data, ReLU activations are used multiple times for their nonlinear nature and relatively high computational efficiency.

The output feature maps of the encoder self-attention mechanism are then passed through the decoder attention. The decoder attention consists of two multi-head attention mechanisms. The first one is used to capture the temporal dependencies within the output sequence, while the second multi-head attention is used to capture the dependencies between the input and the output sequence.

In the decoder’s attention, the output feature maps of the previous layer are used as the input queries, keys, and values. The first multi-head attention outputs the attention weights and the output feature maps, which are then fed through a feed-forward network similar to the encoder feed-forward network. In the second multi-head attention, the output feature maps of the previous layer are used as the input queries, and the encoded input is used as the keys and values. The attention mechanism outputs the attention weights and the output feature maps, which are then added to the output feature maps of the previous layer.

Finally, the output sequence is decoded using a linear layer (Decoder), and the output sequence is projected to the desired length using another linear layer. To summarize, the DFPTransformer network consists of an encoder, a decoder, and multiple attention mechanisms. All the attention mechanisms mentioned before are Multi-head Attention as shown in Fig 1. Residual connection is used in both Encoder Attention and Decoder Attention.

**Fig 2:**
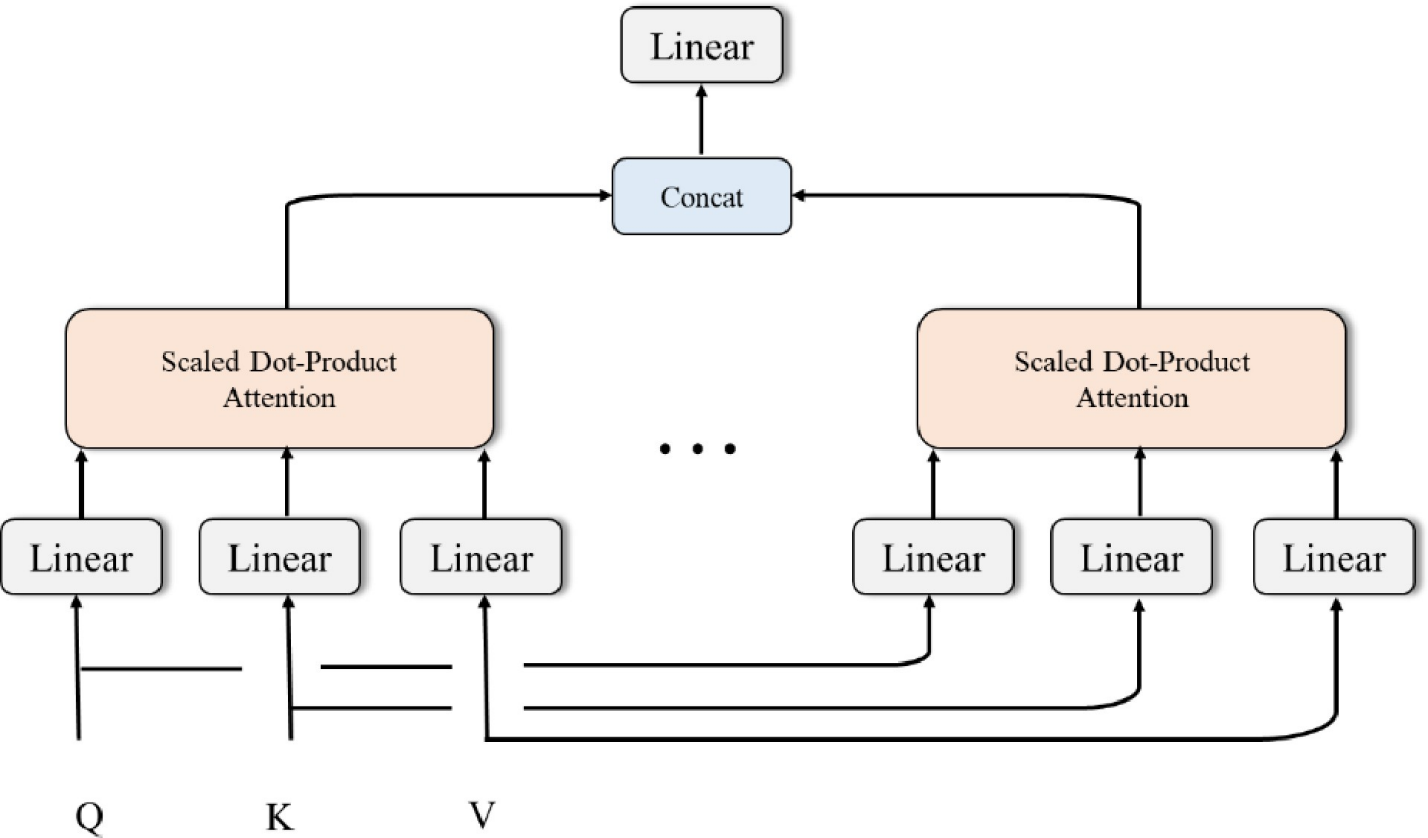
Multi-head attention.

Fig 2 shows a typical Multi-head Attention. Multi-head attention is a powerful mechanism that enables a model to attend to multiple parts of an input sequence simultaneously, allowing it to capture complex patterns and dependencies. The basic idea behind multi-head attention is to project the input sequence into several subspaces and compute attention scores for each subspace separately. The outputs of these subspaces are then concatenated and projected back into the original space, allowing the model to capture diverse patterns and relationships in the input sequence. Formally, given an input sequence X, multi-head attention can be defined as follows:

$$MultiHead(Q,K,V)=Concat({head}_{1},{head}_{2},\ldots ,{head}_{h})W^{o}$$ (1)

where *Q*, *K*, and *V* are the queries, keys, and values, respectively, each with dimensionality *d*_model_. The input sequence is projected into *h* subspaces using separate linear projections:

$${head}_{i}=Attention(QW_{i}^{Q},KW_{i}^{K},VW_{i}^{V})$$ (2)

where Attention is a scaled dot-product attention mechanism, $W_{i}^{Q},W_{i}^{K}$ and $W_{i}^{V}$ are the learnable projection matrices, and *i*∈[1,*h*]. The outputs of the subspaces are then concatenated and projected back into the original space using a learnable projection matrix *W*^*o*^, resulting in the final output of multi-head attention.

The scaled dot-product attention mechanism used in multi-head attention is similar to the one used in standard attention mechanisms, but with an added scaling factor to ensure that the dot product does not get too large. Given a query vector *q*, a key vector *k*, and a value vector *v*, the attention mechanism can be defined as follows:

$$Attention(q,k,v)=softmax(\frac{qk^{T}}{\sqrt{d_{k}}})v$$ (3)

where *d*_*k*_ is the dimensionality of the key vectors. The softmax function is applied over the dot product of the query and key vectors, producing a set of attention scores that indicate the importance of each key vector to the query vector. The value vectors are then weighted by the attention scores and summed to produce the final output of the attention mechanism.

**Fig 3:**
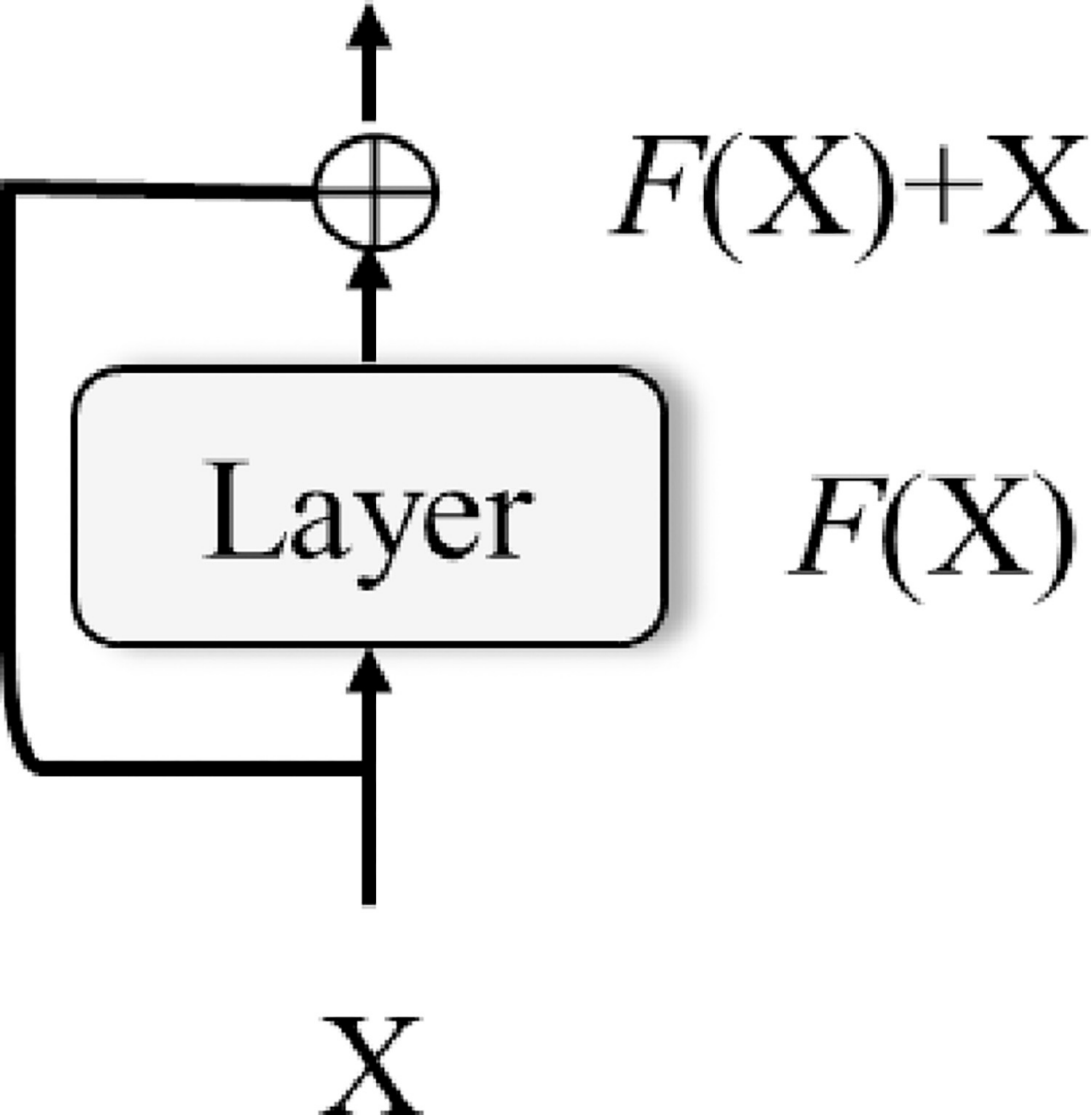
Residual connection.

Fig 3 shows a residual connection. A residual connection is a type of shortcut connection that bypasses one or more layers in a neural network. It involves adding the original input to the output of a layer or block of layers, which helps alleviate the vanishing gradient problem and allows for better training of very deep neural networks. The residual connection can be expressed mathematically as follows:

y=F(X)+X (4)

where *X* is the input to a layer or block of layers, *F* is the transformation performed by the layer, and *y* is the output of the layer with the addition of the original input.

### Data and training approach

The proposed DFPTransformer requires a suitable dataset to be trained effectively. For the problem of foundation pit monitoring, the data should be collected over time to capture both the spatial and temporal correlation between the measurements. Therefore, the dataset should consist of samples distributed along time, where each sample represents a one-dimensional vector of the settlement values of all monitoring points at a given time point. This allows the model to capture the underlying trends and patterns in the data, enabling it to make accurate predictions.

Training a deep learning model requires an optimizer to update the weights and biases of the network during backpropagation. The optimizer adjusts the model parameters to minimize the loss function. The choice of optimizer can significantly affect the training speed and accuracy of the model. In this work, we use the AdamW optimizer, which is a variant of the Adam optimizer [41]. There are two differences between AdamW and Adam: (1) Weight Decay Handling: AdamW separates weight decay from gradient updates, making L2 regularization more accurate and consistent across model parameters, which helps prevent overfitting in deep neural networks; (2) Bias Correction for Learning Rate: AdamW corrects the bias in learning rates, ensuring more stable training by mitigating initial high learning rate issues seen in standard Adam. AdamW has been shown to achieve state-of-the-art performance in various deep learning tasks, including natural language processing and computer vision.

The loss function is a critical component in training a deep learning model. It measures the difference between the predicted output of the model and the actual ground truth. In our work, we use Mean Squared Error (MSE) as the loss function, which is a common choice for regression tasks. MSE calculates the average squared difference between the predicted and actual values. It penalizes large errors more heavily than small errors, making it suitable for applications where accurate prediction is crucial. The MSE loss function can be expressed mathematically as:

$$MSE=\frac{1}{n}\sum_{i=1}^{n}{\left(y_{i}-{\hat{y}}_{i}\right)}^{2}$$ (5)

where *y*_*i*_ is the actual ground truth value, ${\hat{y}}_{i}$ is the predicted value, and *n* is the number of samples in the dataset. The goal of training the model is to minimize the MSE loss by adjusting the model parameters.

## Case study

### Introduction of the foundation pit in the project

**Fig 4:**
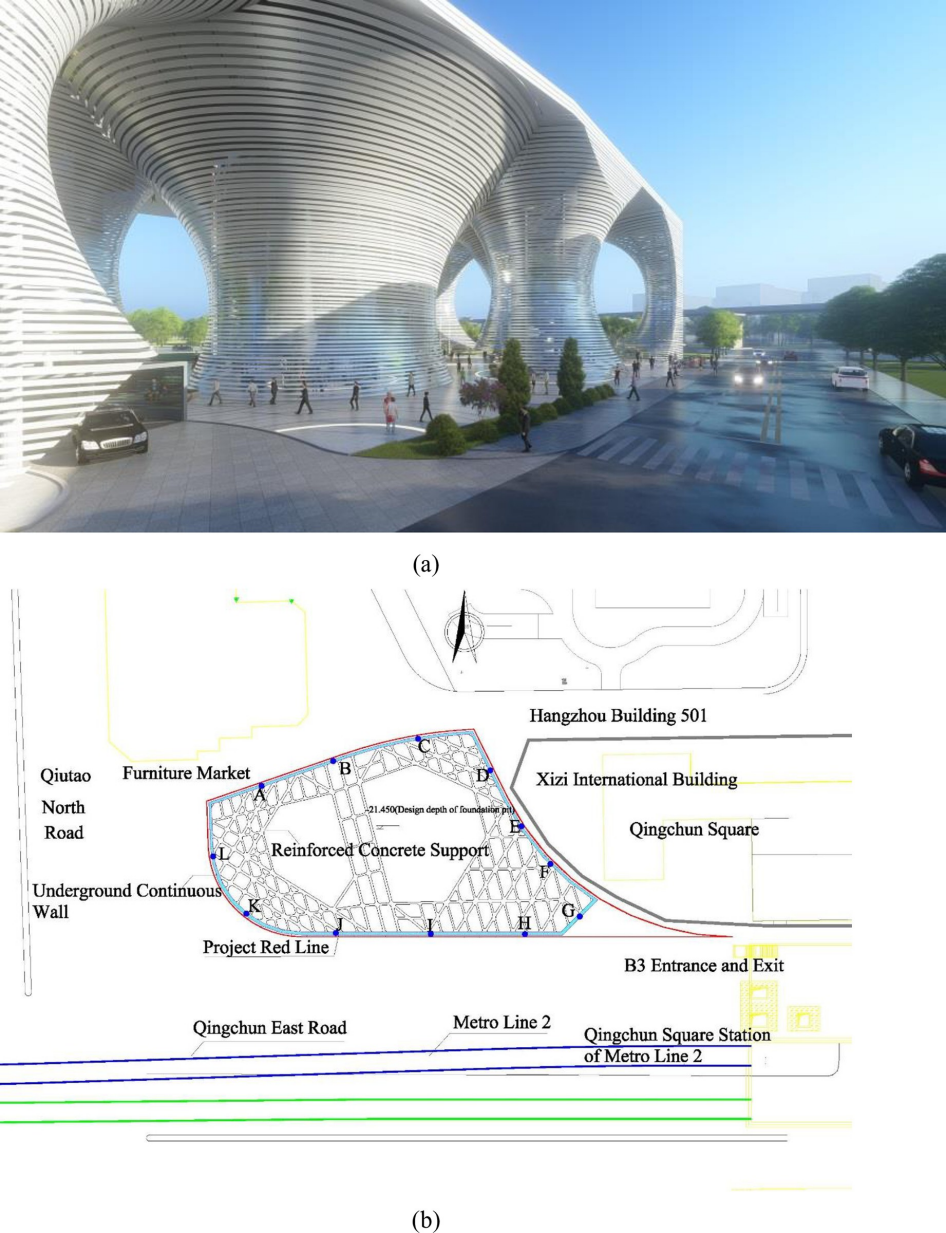
Introduction of foundation pit project: (a) Renderings of the Dragonfly Parking Building Project; (b) Surrounding conditions of the project foundation pit.

As shown in Fig 4, the project used for case analysis is located in Hangzhou City, Zhejiang Province. On the north side of the project, there is a furniture market nearby, which is an early-built shallow foundation structure building, and the deep excavation of the foundation pit will inevitably cause settlement around it. The designed elevation of the pit bottom is -21.45 meters, and the excavation depth is 21.45 meters. The foundation pit support adopts an underground continuous wall combined with four layers of reinforced concrete horizontal internal support. The pit inside the pit is reinforced with high-pressure rotary jet grouting piles for full section reinforcement, and the pit inside is drained and dewatered with pipe wells, while there is no precipitation outside the pit.

During the excavation process of the foundation pit, foundation pit monitoring is used to ensure the safety of the project. The monitoring points are arranged along the foundation pit retaining wall, as shown in Fig 4. Settlement observations are measured using secondary leveling. Observation should adhere to the four fixed principles: the surveyor, the station position, the measuring instrument, and the surveying order to ensure the quality of observation data. The monitoring personnel record deformation data and other information in the daily monitoring report. The data in this paper are extracted from the daily complete monitoring report of the foundation pit, with a total of 220 samples.

### Process of the data

After collecting the dataset, the report of the foundation pit has two types of settlement: single-day deformation and cumulative deformation. Through simple visualizations, it can be observed that single-day deformation does not have a clear pattern, while cumulative deformation follows a certain pattern. Therefore, this study uses cumulative deformation as the indicator for training and prediction, and previous research has also adopted cumulative deformation (Zhang et al., [20]).

**Fig 5:**
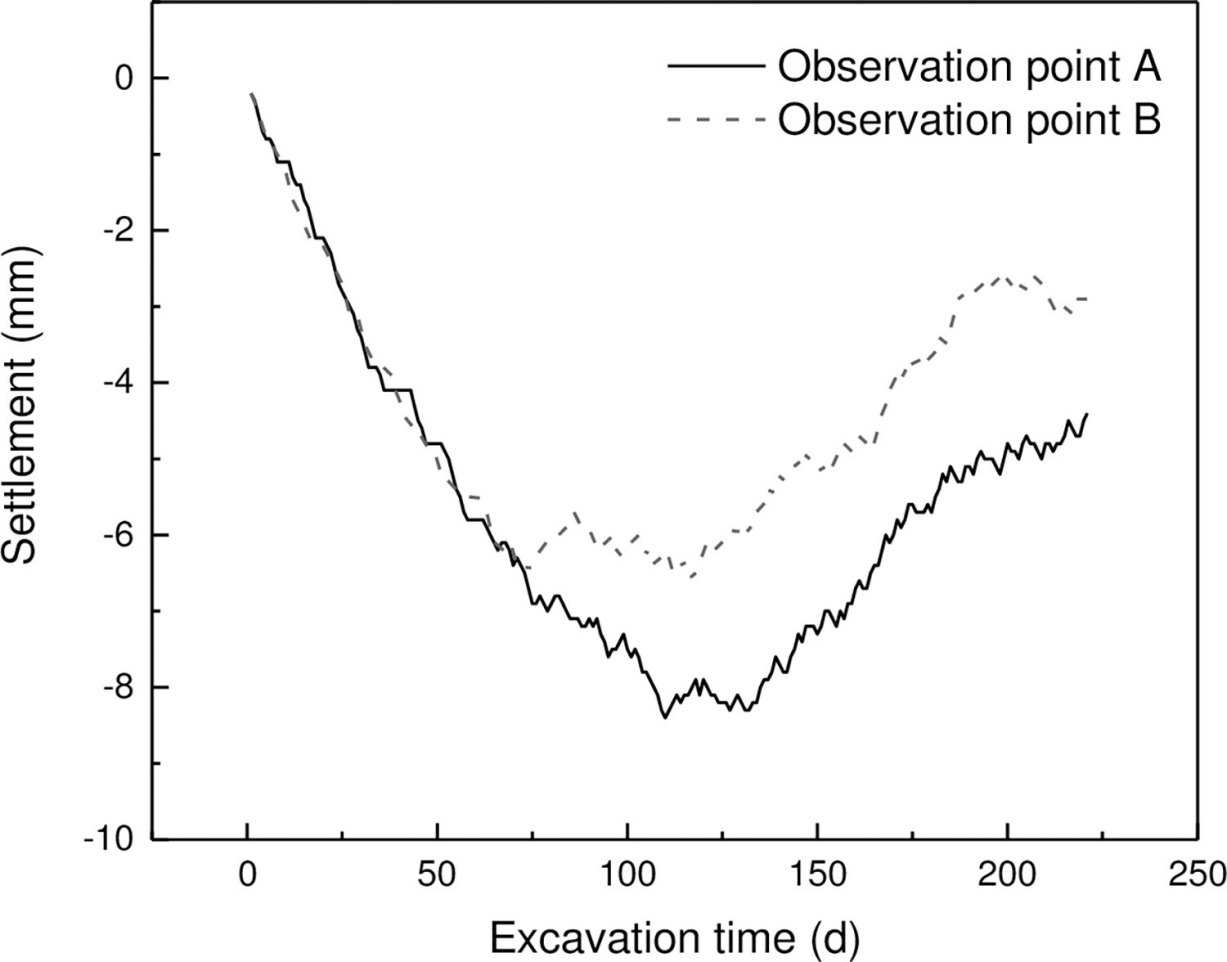
Distribution of settlement of foundation pit over time.

Fig 5 shows the displacement of ground settlement at two monitoring points over time. The horizontal axis represents the excavation time in days, and the vertical axis represents the settlement in millimeters. It can be observed that: (1) the curve is generally negative, indicating settlement; (2) the curve shows a trend of first declining and then rebounding, (3) the settlement values are generally distributed between 0 and -10mm.

**Fig 6:**
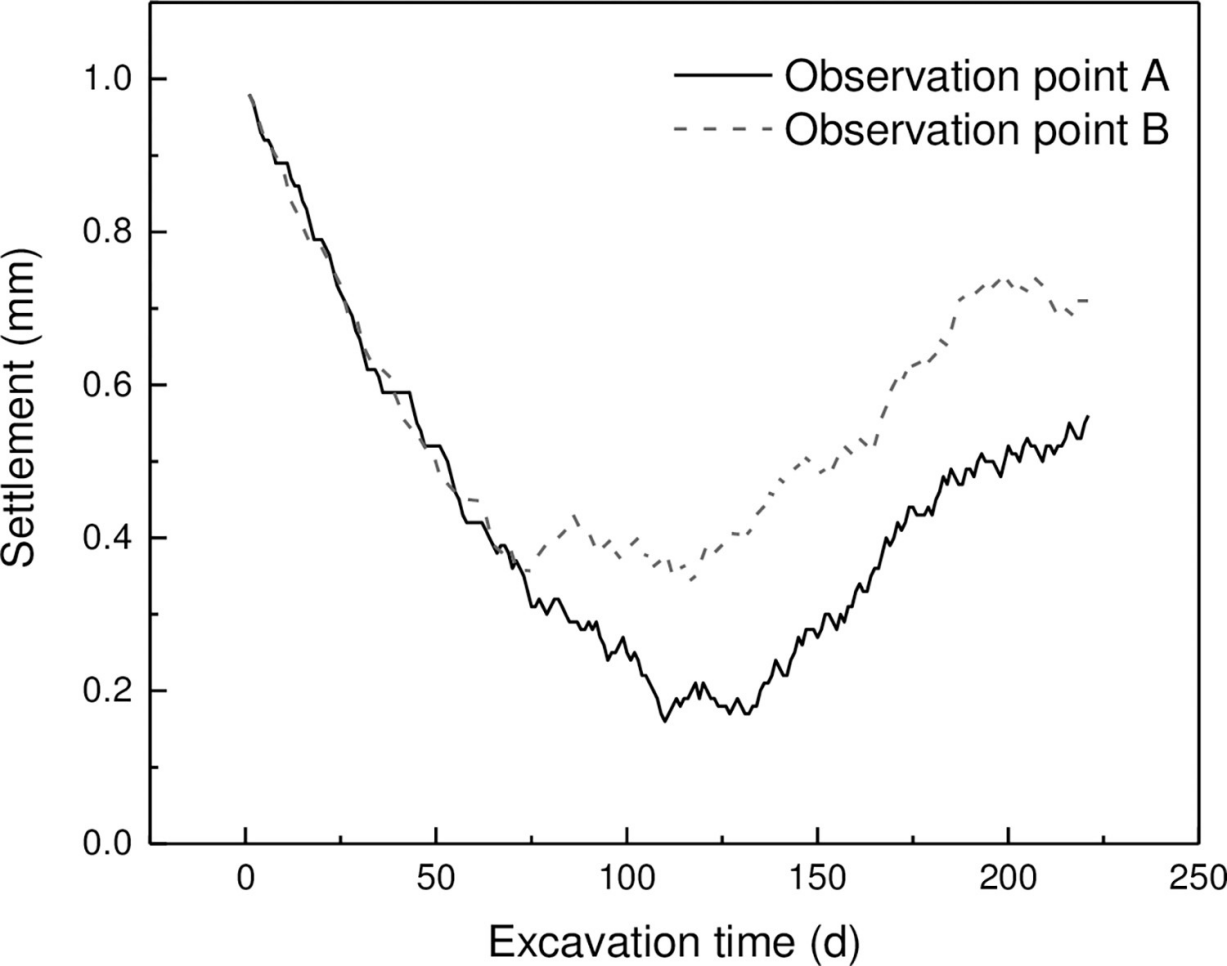
Normalized time series settlement of foundation pit.

In deep learning training, data normalization is very helpful for model training. Min-max normalization is a common data normalization technique used in machine learning to rescale the input data to a common range. It involves scaling the original data between 0 and 1. The formula for min-max normalization is:

$$X\_norm=\frac{\left(X-X_{min}\right)}{X\_\max-X\_min}$$ (6)

where *X* is the original data point, *X*_*min* is the minimum value of the dataset, and *X*_*max* is the maximum value of the dataset. The resulting normalized value *X*_*norm* represents the original value’s relative position within the range of the dataset. This normalization method is simple and effective in improving model performance. In this dataset, the minimum limit is set at -10.0 mm and the maximum limit is set at 0 mm. Fig 6 shows the results after normalization, and it can be observed that the data is distributed between 0 and 1.0.

The single data sample is generated following the requirement of the deep learning model. At any given time, this study arranges all the settlement values of the monitoring points in a column vector according to their spatial order, so that the model can take into account the correlation between all monitoring points. In addition, considering the temporal correlation, the settlement prediction is treated as a time series prediction problem, and the monitoring data of *sequence_in* days is used to predict the settlement monitoring value of the next day. Thus, as shown in the Table 1, the dimension of a single sample input is [*sequence_in*, *num_observation*]. Where the *num_obsevation* is the number of the monitoring points. The dataset is divided into training and testing sets. There are a total of 220 days of monitoring data, and the training set and testing set are divided in a ratio of 0.8. Considering the number of days for a single sample is (*sequence_in+*1), there are 170 samples in the training set and 39 in the testing set, where *sequence_in* is set as 6. The size is almost the same as that in the previous research (such as Zhang et al. [20]).

**Table 1:** Input and output of the deep learning model.

	shapes	Description
**Input**	[*sequence_in*, *num_observation*]	Settlement vectors in multiple time steps.
**Output**	[1, *num_observation*]	Settlement vectors in the next time steps.

### Model training

Overfitting and underfitting are common problems in machine learning models. Overfitting occurs when the model is too complex and fits too closely to the training data, leading to poor generalization of new data. On the other hand, underfitting occurs when the model is too simple and fails to capture the underlying patterns in the data, resulting in high bias and poor performance on both training and test data.

**Fig 7:**
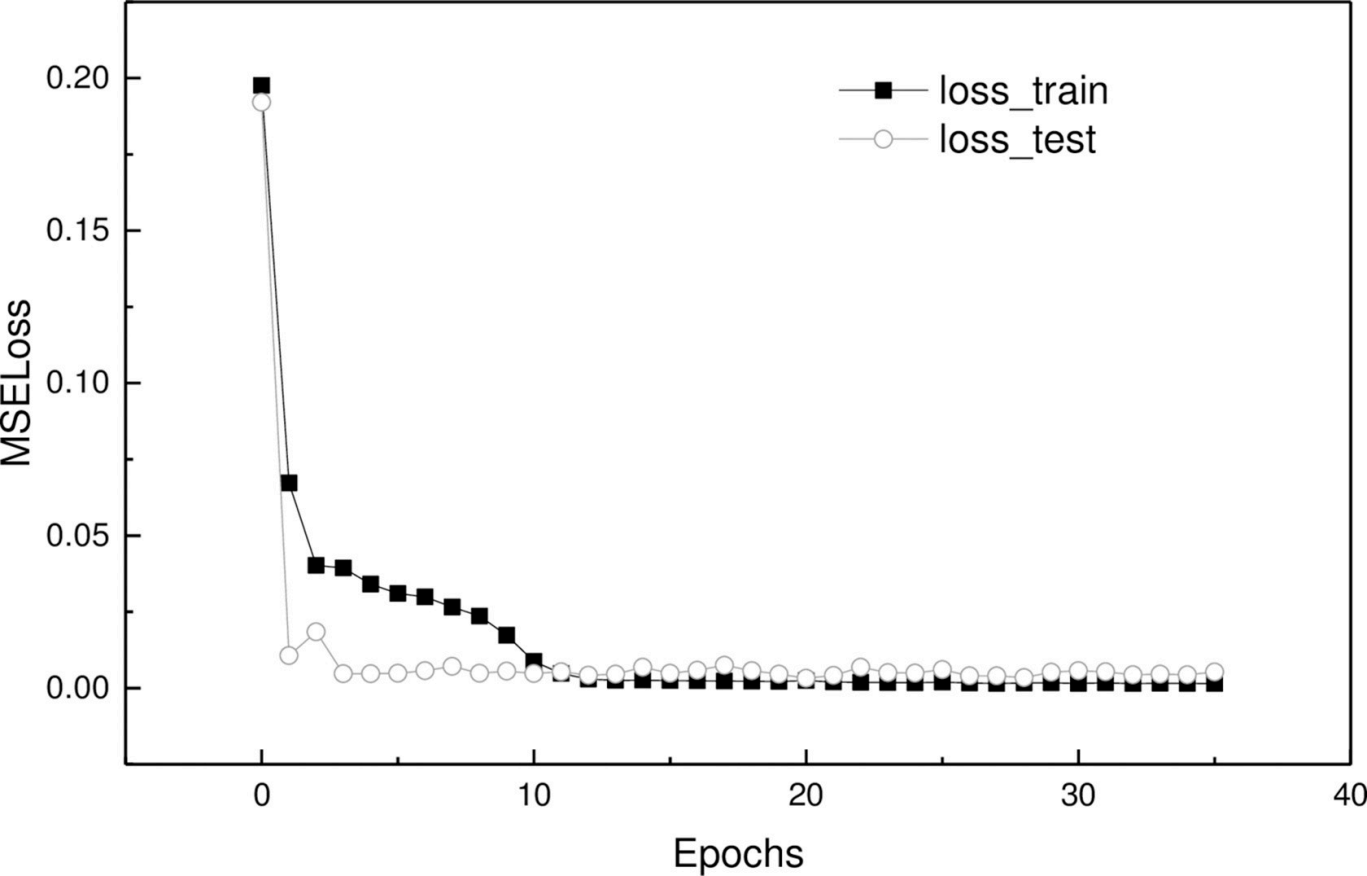
Training loss descent process.

Fig 7 shows the training loss descent process during the training of the proposed model. The training loss decreases continuously during the training process, indicating that the model is learning to fit the training data. The test loss, which measures the model’s performance on the test data, also decreases initially and makes small fluctuations after reaching the minimum value, indicating that the model is not overfitting excessively.

To prevent overfitting, the paper employs an early-stopping strategy. The model is trained for a fixed number of epochs, and the training is stopped when the performance on the test set does not improve for a certain number of epochs. This strategy prevents the model from continuing to learn the noise in the training data, which leads to overfitting. By stopping the training at the point where the performance on the validation set is the best, the proposed model achieves good performance on the test data.

### Model performance evaluation

#### Evaluation on metrics

After training the model on the training set, it is important to evaluate the model’s performance on the test set to assess its generalization capability. This paper uses two indicators, absolute error (AE) and absolute error percent (APE), to evaluate the model’s performance. Absolute error is a measure of the difference between the predicted value and the true value. It is calculated as the absolute difference between the predicted value ${\hat{y}}_{i}$ and the true value *y*_*i*_:

$$AE=\frac{1}{n}\sum_{i=1}^{n}\left|y_{i}-{\hat{y}}_{i}\right|$$ (7)

Absolute error percent is a relative measure of the difference between the predicted value and the true value. It is calculated as the absolute difference between the predicted value ${\hat{y}}_{i}$ and the true value *y*_*i*_, divided by the true value *y*_*i*_:

$$APE=\frac{1}{n}\sum_{i=1}^{n}|y_{i}-{\hat{y}}_{i}|/y_{i}$$ (8)

Both AE and APE provide information about the accuracy of the model’s predictions. AE measures the magnitude of the error in the predicted values, while APE provides a relative measure of the error. By using these two indicators, one can quantitatively assess the model’s performance and compare it to other models. The goal is to minimize both AE and APE, indicating that the model is accurately predicting the behavior of the foundation pit.

Table 2 shows the model’s performance on two evaluation metrics, absolute error, and absolute error percent, compared with Zhang et al.’s [20] results. The results demonstrate that our proposed approach outperforms Zhang et al.’s method in terms of absolute error, with a performance of 0.40mm compared to their best result of 0.59mm. However, our method has a slightly worse performance in absolute error percent, with a performance of 8.61% compared to Zhang et al.’s 7.53%. It is important to note that Zhang et al. [20] used a variety of information in their method, while our approach only utilized settlement data. This further highlights the advantages of our approach in settlement prediction.

**Table 2:** Average prediction errors of DPFTransformer.

Method	AE (mm)	APE(%)
**Ours**	0.40	8.61
**Zhang et al. [20]**	0.59	7.53

The success of our approach can be attributed to several factors. Firstly, this paper adopts an attention mechanism, which is a key factor that contributes to superior performance. The attention mechanism is used to learn the importance of each monitoring point in the prediction of settlement, allowing the model to focus more on the relevant points and less on the irrelevant ones. This mechanism helps to improve the model’s prediction accuracy and ensure that the model is more robust.

Secondly, this paper fully considers the spatial correlation among monitoring points, which is another key factor that contributes to superior performance. Settlement prediction is a spatial-temporal problem, and the correlation among monitoring points in the same location and different locations plays a crucial role in the accuracy of the prediction. The proposed approach takes into account the spatial correlation among monitoring points, enabling the model to capture the inherent characteristics of the project and improve prediction accuracy.

Moreover, this paper also applies the early-stopping mechanism and dropout regularization to avoid overfitting and improve the robustness of the model. The early-stopping mechanism stops the training process when the model starts to overfit on the training set, preventing the model from memorizing the training data and making accurate predictions on new data. The dropout regularization technique reduces the interdependent learning between neurons and increases the independence of the model’s parameters, making the model more robust and avoiding overfitting.

#### Parameter sensitivity analysis

**Fig 8:**
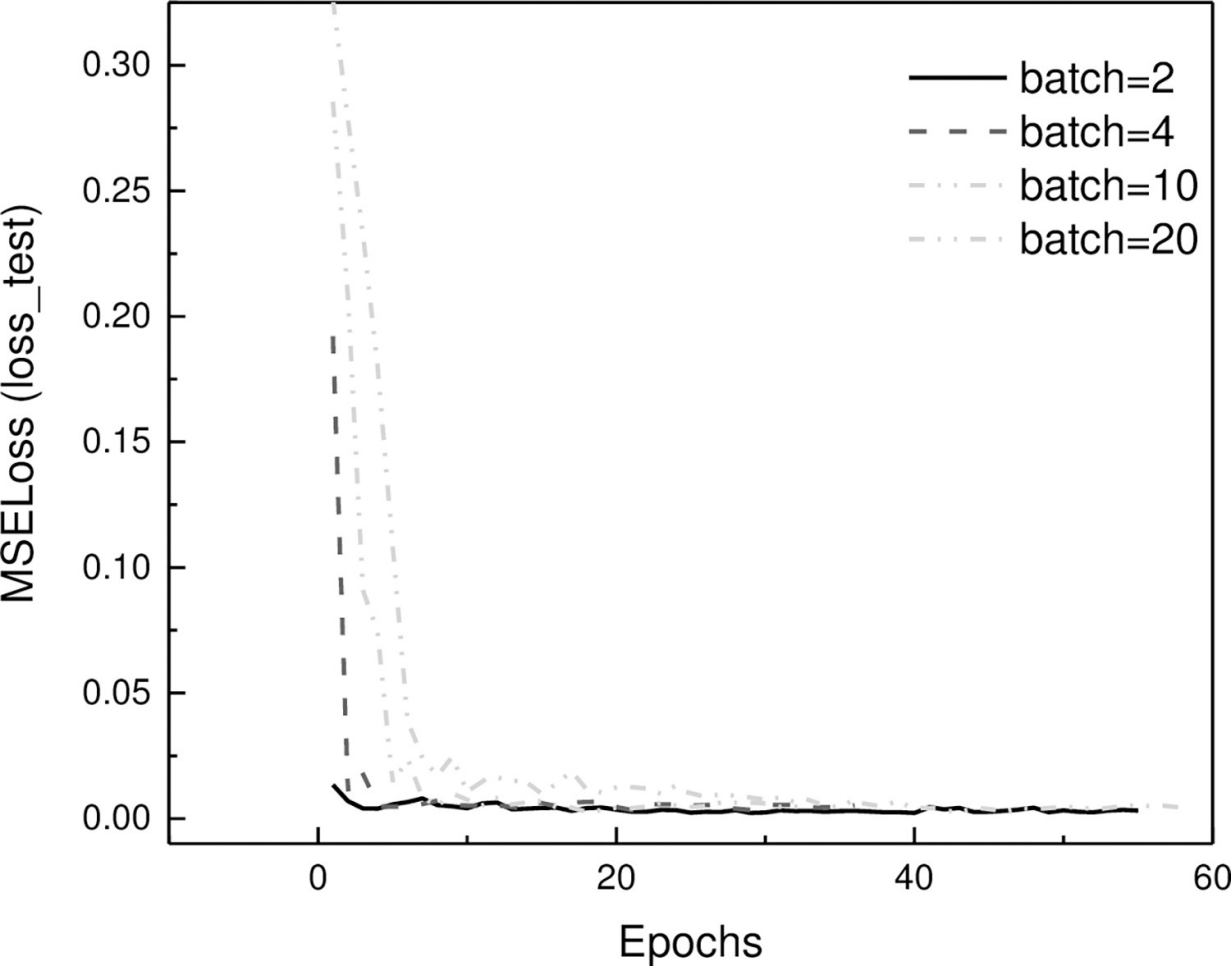
Influence of batch size on training loss on test set.

Parameter sensitivity analysis investigates how variations in model parameters impact its performance. The influence of different batch sizes on the DFPTransformer model’s performance. Multiple experiments are conducted to evaluate the parameter sensitivity of the proposed model. Fig 8 shows the test loss descent process under different batch sizes. The results indicate that the performance of the model is not significantly affected by the batch size.

#### Model sensitivity and uncertainty analysis

The analysis of model sensitivity and uncertainty aims to assess the model’s capability to excel when handling data that deviates from the training data, such as data with noise or corruption. In this specific study, a dataset (DATASET1) was formed by arranging settlement data for all monitoring points in a sequential manner. Its performance underwent a quantitative evaluation based on the metrics presented in Table 1. Furthermore, in order to validate the model’s sensitivity and resilience to uncertainty, an additional dataset (DATASET1_REVERSE) was generated by reversing the vectors within the original dataset. The comparison in Table 3 between the model’s performances on these two datasets reveals that the model exhibits identical performance on both. This serves as evidence of the model’s robustness.

**Table 3:** Model sensitivity of DPFTransformer.

Dataset	AE (mm)	APE(%)
DATASET1	0.40	8.61
DATASET1_REVERSE	0.40	8.61

## Result and discussion

**Fig 9:**
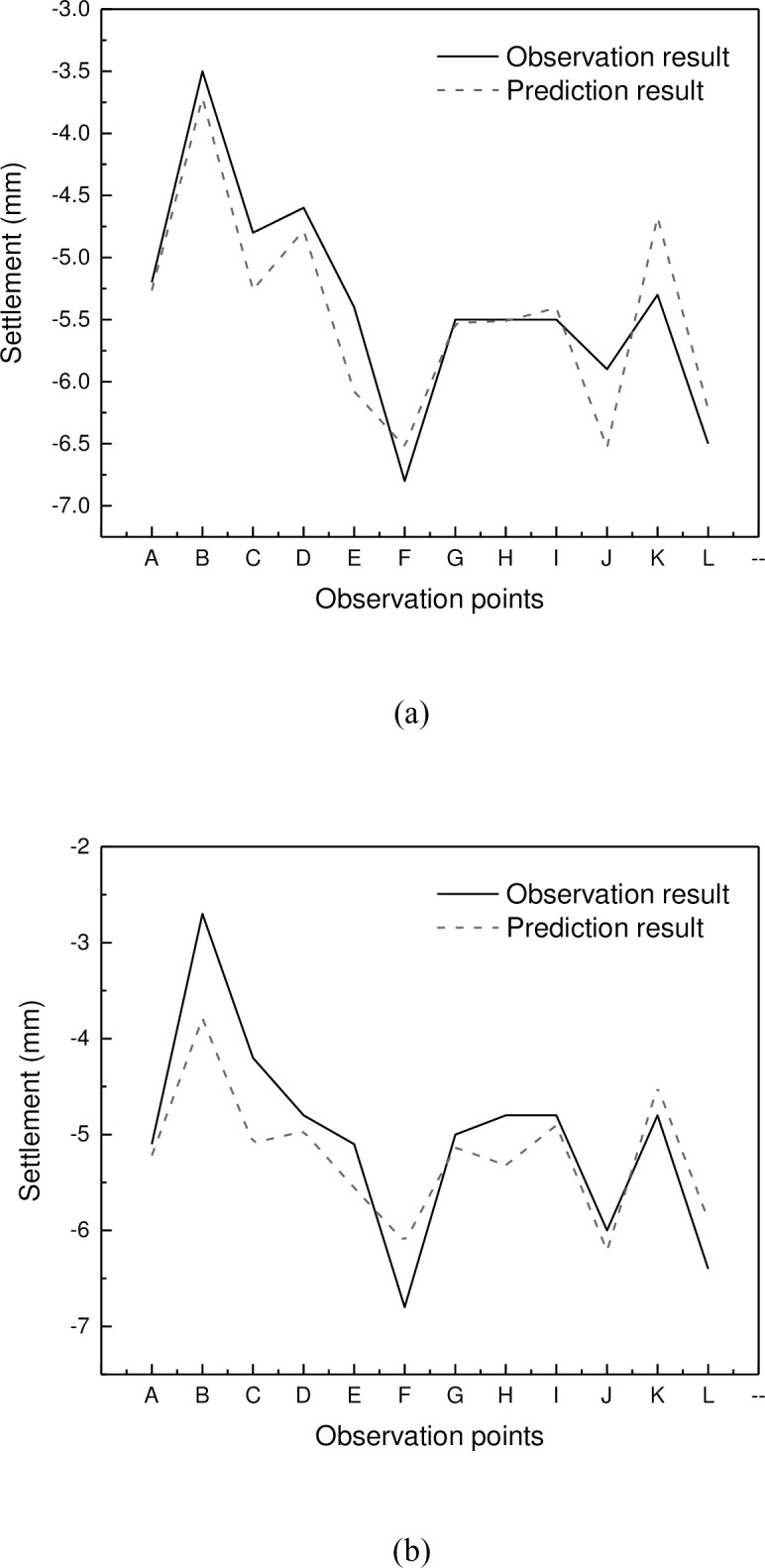
Prediction result distributed over observation points: (a) time 1; (b) time 2.

Fig 9 shows the prediction result distributed over observation points. It can be seen that the spatial variation of the two time points is generally predicted with a small error in several observation points. This indicates that the model is capable of effectively learning the spatial correlation between the different observation points, as well as the temporal changes in the settlement of the foundation pit.

To fully validate the importance of the spatial relation, a basic LSTM network without considering the spatial correlation is trained and compared with the proposed DFPTransformer. The LSTM model presented is a simple recurrent neural network architecture that uses an LSTM layer followed by a fully connected layer. The dropout rate is also used to prevent overfitting during training. Besides, a similar RNN-LSTM based hybrid model is also trained for comparison. As there is no spatial correlation, the dataset is just a settlement of every separate single monitoring point.

**Fig 10:**
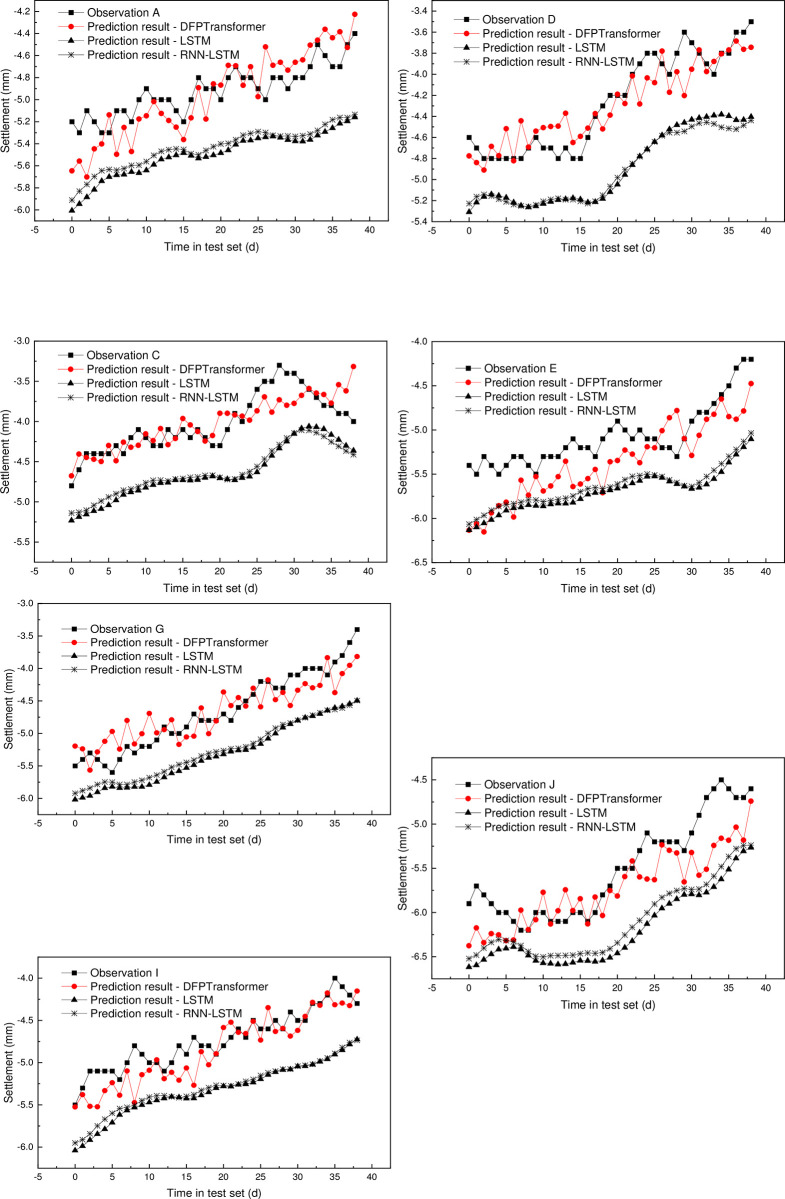
Prediction result compared with LSTM.

Fig 10 presents the comparison between the real curve, the prediction of the DFPTransformer network, and the other two networks for various observation points. The results indicate that the proposed DFPTransformer network is capable of accurately predicting the daily settlement, which is very close to the real curves. It can be observed that the predicted curves of the DFPTransformer network are more similar to the real curves as compared to those of the LSTM network and the RNN-LSTM network. The significant differences result from (1) The LSTM-based method didn’t take into account the spatial correlation, which is crucial. (2) The LSTM-based method did not include various components proposed by our model and is relatively simple. (3) The Transformer model has proved to be better than the LSTM model in many tasks.

These findings suggest that the consideration of spatial correlation is crucial and can help predict the settlement of deep foundation pit. Besides, the attention mechanism used in the DFPTransformer network is found to be useful in improving the prediction result. The attention mechanism enables the model to focus on specific areas that require more attention during the prediction process, allowing the model to capture the spatial correlation between observation points effectively. This is due to the ability of the attention mechanism to weigh the contribution of each observation point to the prediction result based on its importance, allowing the model to assign higher weights to the more significant observation points. Therefore, the DFPTransformer network can accurately predict the daily settlement of deep foundation pits with a high level of precision.

**Fig 11:**
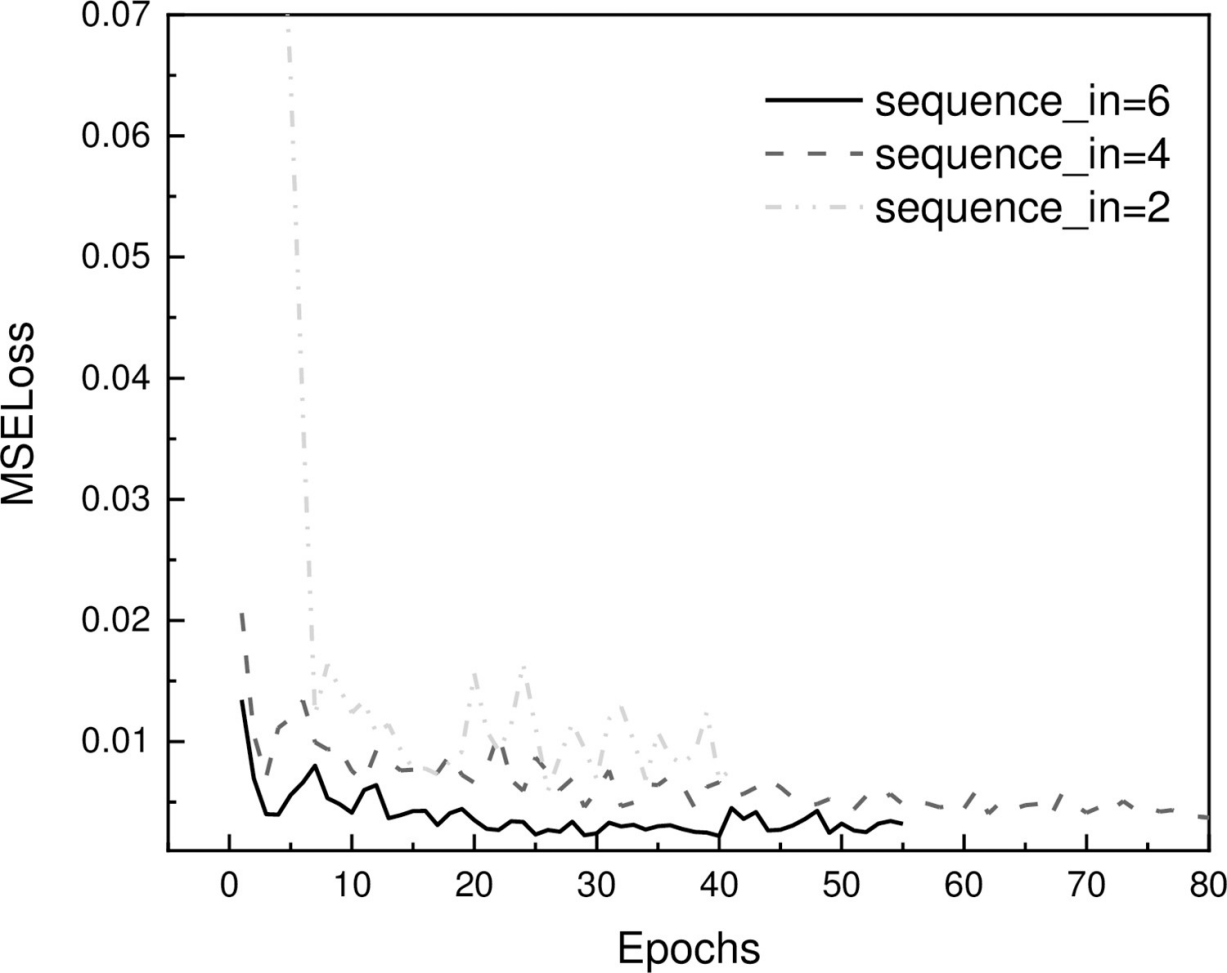
Influence of sequence_in on the prediction result.

Moreover, the *sequence_in* parameter plays a crucial role in the proposed approach. As demonstrated in Fig 11, the influence of *sequence_in* on the prediction result is illustrated. As the *sequence_in* value increases, the MSE loss decreases slightly. However, the results also indicate that the change in *sequence_in* does not lead to a significant change in the MSE loss. The reason why the *sequence_in* value is set to 6 in this study is mainly due to the fact that increasing *sequence_in* would greatly reduce the available data size. Since the data used in this study is from the construction process of a deep foundation pit, which is highly valuable and not abundant, the *sequence_in* value is selected carefully to ensure that a sufficient amount of data is used.

## Conclusions

In summary, this paper proposes a deep learning method DPFTransformer that can consider both spatial and temporal correlations among monitoring points in excavations, and use the attention mechanism to handle the spatial correlations among excavation monitoring points and incorporate temporal sequence information for prediction. The main conclusions are:

1. The DPFTTransformer with spatial correlation dataset outperforms LSTM without spatial correlation, demonstrating the significance of spatial correlation for predicting the settlement of deep foundation pit accurately;
2. A performance of our 0.40mm compared to Zhang et al.’s [20] 0.59mm in AE, showing that the attention mechanism is useful in handling the temporal correlations and spatial correlations among monitoring points;
3. the proposed approach is capable of predicting the settlement of the deep foundation pit more accurately and can serve as a practical tool in the deep foundation pit construction process.

In addition to the conclusions drawn from this study, there are several potential avenues for future research in the field of intelligent geotechnical engineering. One notable area is the extension of our approach to investigate the effectiveness of predicting settlement in other types of foundation pits, such as square or rectangular ones. This would involve adapting the proposed model to capture the specific characteristics and challenges associated with different pit geometries.
